# Differences in the distribution, phenotype and gene expression of subretinal microglia/macrophages in C57BL/6N (Crb1^rd8/rd8^) versus C57BL6/J (Crb1^wt/wt^) mice

**DOI:** 10.1186/s12974-014-0221-4

**Published:** 2015-01-15

**Authors:** Bogale Aredo, Kaiyan Zhang, Xiao Chen, Cynthia Xin-Zhao Wang, Tao Li, Rafael L Ufret-Vincenty

**Affiliations:** Department of Ophthalmology, UT Southwestern Medical Center, 5323 Harry Hines Blvd, Dallas, TX 75390-9057 USA; Current address: Department of Ophthalmology, Hainan Provincial People’s Hospital, Haikou, Hainan 570203 PR China; Current address: Department of Ophthalmology, Wuhan General Hospital of Guangzhou Military Command, Wuhan, 430070 PR China

**Keywords:** subretinal, macrophages, microglia, rd8, CD16, Iba-1, aging, gene expression, activation, *Crb1*

## Abstract

**Background:**

Microglia/macrophages (MG/MΦ) are found in the subretinal space in both mice and humans. Our goal was to study the spatial and temporal distribution, the phenotype, and gene expression of subretinal MG/MΦ in mice with normal retinas and compare them to mice with known retinal pathology.

**Methods:**

We studied C57BL/6 mice with (C57BL/6N), or without (C57BL/6J) the rd8 mutation in the *Crb1* gene (which, in the presence of yet unidentified permissive/modifying genes, leads to a retinal degeneration), and documented their fundus appearance and the change with aging. Immunostaining of retinal pigment epithelium (RPE) flat mounts was done for 1) Ionized calcium binding adaptor (Iba)-1, 2) FcγIII/II Receptor (CD16/CD32, abbreviated as CD16), and 3) Macrophage mannose receptor (MMR). Reverse-transcription quantitative PCR (RT-qPCR) was done for genes involved in oxidative stress, complement activation and inflammation.

**Results:**

The number of yellow fundus spots correlated highly with subretinal Iba-1+ cells. The total number of subretinal MG/MΦ increased with age in the rd8 mutant mice, but not in the wild-type (WT) mice. There was a centripetal shift in the distribution of the subretinal MG/MΦ with age. Old rd8 mutant mice had a greater number of CD16+ MG/MΦ. CD16+ cells had morphological signs of activation, and this was most prominent in old rd8 mutant mice (*P* <1×10^−8^ versus old WT mice). Subretinal MG/MΦ in rd8 mutant mice also expressed iNOS and MHC-II, and had ultrastructural signs of activation. Finally, rd8 mutant mouse RPE/ MG/MΦ RNA isolates showed an upregulation of *Ccl2*, *CFB*, *C3*, *NF-kβ*, *CD200R* and *TNF-alpha*. The retinas of rd8 mutant mice showed upregulation of *HO-1*, *C1q*, *C4*, and *Nrf-2*.

**Conclusions:**

When compared to C57BL/6J mice, C57BL/6N mice demonstrate increased accumulation of subretinal MG/MΦ, displaying phenotypical, morphological, and gene-expression characteristics consistent with a pro-inflammatory shift. These changes become more prominent with aging and are likely due to the combination of the rd8 mutation and yet unidentified permissive/modulatory genes in the C57BL/6N mice. In contrast, aging leads to a scavenging phenotype in the C57BL/6J subretinal microglia/macrophages.

**Electronic supplementary material:**

The online version of this article (doi:10.1186/s12974-014-0221-4) contains supplementary material, which is available to authorized users.

## Background

Microglia are the resident macrophages of the central nervous system (CNS). The blood brain barrier and the blood retina barrier do not allow the immune system an open communication to the brain and retina respectively; this is an important element of the immune privilege in the CNS. Yet, these tissues have an extremely delicate homeostasis that needs to be maintained. Microglia are the resident immune cells that constantly patrol/scavenge the CNS, including the retina, to detect any pathologic state (for example, damaged neurons, extracellular debris and infectious agents) that would require a response.

Pathological activation of microglia may play an important causative role in Alzheimer’s disease and other neurodegenerative diseases [1–3]. Since the retina is an extension of the brain, it seems reasonable to predict that activated microglia may also be at play in degenerative retinal diseases. The relevance of microglia/macrophages (MG/MΦ) in age-related macular degeneration (AMD) is supported by their presence in a very high percentage of both neovascular and geographic atrophy specimens in human AMD cases [4–6]. In the Submacular Surgery Trial [7] about 80% of excised AMD-related choroidal neovascular lesions contained macrophages. More recently, microglia have been reported in the subretinal space of patients with retinitis pigmentosa and AMD [8]. Combadiere *et al*. found subretinal microglia in patients with AMD, but not age-matched controls [9]. The cells were found in areas of localized retinal pigment epithelium (RPE) disruption and/or photoreceptor degeneration.

Subretinal MG/MΦ also appear to be important in responding to retinal pathology in mice and other mammals [9–14]. Accumulation of subretinal MG/MΦ has been described in multiple models of acute and chronic retinal pathology including increased light exposure [11], blue light injury [15], laser-induced choroidal neovascularization [16], and retinal degenerations [17,18]. Manipulating the receptors and ligands involved in microglial migration (for example, *CX3CR1*, *Ccr-2*, and *Ccl-2*) can also lead to an increased accumulation of subretinal MG/MΦ [13,19,20]. Recently we described the generation of complement factor H transgenic mice that develop early signs of AMD, including the early accumulation of basal laminar deposits [21]. Interestingly, these mice also develop increased accumulation of subretinal MG/MΦ in the central retina, despite the fact that we have not manipulated any genes directly involved in the regulation of macrophage trafficking and that they do not express the recently described rd8 mutation. The rd8 mutation is a single nucleotide deletion in the *Crb1* gene that has been found in many strains of mice, and leads to disruption of the external limiting membrane of the retina [22,23]. In the presence of yet undefined permissive/modulatory genes, the rd8 mutation also results in a multifocal retinal degeneration, and the accumulation of subretinal MG/MΦ [22,24]. Luhmann *et al*. have recently identified a candidate region on chromosome 15 that may carry one or more of these modifiers [25].

The role of subretinal MG/MΦ in maintaining homeostasis and/or inducing disease is not well understood. With this in mind, we decided to study the natural history of subretinal MG/MΦ in C57BL/6 mice, either in the presence (Group 1; referred to as Crb1^rd8/rd8^, or ‘rd8/rd8’, or ‘rd8 mutant’, or C57BL/6N), or absence (Group 2; referred to as Crb1^wt/wt^, or ‘wt/wt’, or ‘WT’, or C57BL/6J) of the *Crb1* rd8 mutation. We first looked at the changes with normal aging in the number, distribution, and phenotype/morphology of subretinal MG/MΦ in C57BL/6N versus C57BL/6J mice. We also analyzed the expression of complement activation and inflammation-related genes in the retina and in the RPE/subretinal MG/MΦ in both groups.

## Methods

### Animals

C57BL/6 (B6) mice of different ages that were either homozygous (C57BL/6N from Charles River Labs), or wild-type (WT) (C57BL/6J from The Jackson Lab and the National Eye Institute (NEI) aging mouse colony) for the rd8 mutation in the *Crb1* gene were used for this study. There was no difference in the number of fundus spots or the number of ionized calcium binding adaptor (Iba)-1+ subretinal MG/MΦ for mice in the 2 to 4mo versus 5 to 8mo age ranges, or in the 14 to 16mo versus 17 to 20mo age ranges, so mice were age-matched into two groups: 2- to 8-month-old (classified here as ‘young’) and 14- to 20-month-old (classified here as ‘old’). Specifically, for the experiments involving immunohistochemistry (IHC) of RPE-flat mounts we used: young mice (wt/wt or WT, n = 10; rd8/rd8, n = 8), and compared them to old mice (wt/wt, n = 13; rd8/rd8, n = 11). For the qPCR experiments we used 6- to 8-month-old C57BL6/J (wt/wt) mice from The Jackson Lab (n = 11) and age-matched C57BL/6N (rd8/rd8) mice from Charles River Labs (n = 11). Genotyping for the rd8 mutation was done using the primers and protocol previously described [23]. Sequencing for the rd8 mutation was done after a PCR reaction using the primers and protocol described before [13]. All vendor animals were acclimated to our animal facility for at least 1 month before the experiments. The mice were kept in a barrier animal facility at UT Southwestern Medical Center under normal lighting conditions with 12 h-on/12 h-off cycles. The average intensity inside the cages was 40 lux. Most retina laboratories consider normal lighting conditions to be 100 to 500 lux outside the cages [26,27], but less than 100 lux inside the cages [28,29]. All experiments were performed in compliance with the National Institutes of Health (NIH) Guide for the Care and Use of Laboratory Animals, and approved by the UT Southwestern Medical Center Institutional Animal Care and Use Committee (IACUC). Animals were anesthetized one at a time before any procedures in order to prevent opacification of the media. A ketamine-xylazine cocktail was used (100 mg/kg ketamine, 5 mg/kg xylazine).

### Fundus photography and fundus yellow spot counting

Eyes were dilated using one drop per eye of a tropicamide 1% solution (Alcon laboratories, Inc., Fort Worth, TX, USA) 5 minutes before taking retinal images. A Micron III rodent retinal imager (Phoenix Research Laboratories, Pleasanton, CA, USA) was used to take bright field images of the retinas. The yellow spots in either only the central fundus (a circle with a radius equal to 5 disc diameters and centered on the optic nerve), or in the entire fundus (including all peripheral directions up to the ora serrata) were counted. For the central counting, all images were analyzed under identical parameters, and a transparent plastic film marked with a circle having a 5 disc diameter (DD) radius was placed over the fundus photographs and centered on the optic nerve in order to standardize the area to be counted.

### Preparation of retinal pigment epithelium-choroid-sclera flat mounts and immunohistochemistry

Eyes were enucleated, including the nictitating membrane or ‘third eyelid’. This ‘third eyelid’ marked the nasal aspect of the eye, allowing us to preserve the information regarding orientation throughout the processing of the eyes. The eye balls were placed in 4% paraformaldehyde for 2 hours at room temperature (RT). After two washes in phosphate buffered saline (PBS), the removal of the anterior segment was done in a modified way that allowed us to preserve the entire posterior cup. We did this by cutting the eye anterior to the limbus. The iris was removed after confirming that we had preserved the posterior segment all the way to the ora. The retina was then removed from the RPE-choroid eyecup. The remaining posterior eye cup (RPE-choroid-sclera complex, here referred to as ‘RPE flat mount’ or ‘flat mount) was flattened by making 4 to 6 long radial cuts. The RPE flat mounts were incubated in a blocking buffer of 5% bovine serum albumin in 1X PBS (w/v) containing 0.3% (v/v) Triton X-100 for 2 hours at RT. After removing the blocking buffer, the flat mounts were incubated at 4°C overnight with primary antibodies prepared in a diluted (1:5) blocking buffer. The flat mounts were single, double, or triple stained for Iba-1, mouse Macrophage Mannose Receptor (MMR), and/or mouse FcγIII/II Receptor (CD16/CD32, abbreviated as CD16). The antibodies and dilutions used were: rabbit anti-Iba-1 (1:500), goat anti-MMR (1:50) and rat anti-CD16 (1:25) (see Additional file 1: Table S1). The next day, flat mounts were washed 3 × 10 min in 1X PBS and incubated with fluorophore-conjugated secondary antibodies (1:200 dilution) for 2 h at room temperature. The secondary antibodies for double staining experiments included AF 594 donkey anti-rabbit, which was combined with either AF488 donkey anti-goat or AF488 donkey anti-rat. For triple staining experiments we used CF750 donkey anti-rabbit, AF 594 donkey anti-goat, and AF 488 donkey anti-rat. After three 10-min washes in 1X PBS, the flat mounts were cover-slipped and mounted with Prolong Gold antifade reagent with DAPI (double staining experiments) or without DAPI (triple staining experiments).

### Imaging and microglia/macrophage counting on retinal pigment epithelium flat mounts

The flat mounts were photographed using a Zeiss AxioObserver motorized wide-field epifluorescence microscope equipped with a Hamamatsu Orcall-BT-1024G monochrome camera and fluorescence filter sets for FITC, Texas Red, and CY7. Each flat mount was imaged using Zeiss Axiovision software under two or three channels, depending on the experiment, using the 10X objective and 1.6x optivar. A set of images per location, that is, a photographic field, in a flat mount were opened in Adobe Photoshop and the cells were circled and counted for each channel.

### Measurement of microglial/macrophage morphology activation parameters

Microglia/macrophages on flat mounts (n = 3 to 5 eyes per group) that were triple stained for Iba-1, MMR and CD16 were measured as follows: 3 to 5 snapshots/photographic field images from each flat mount were opened in ImageJ software (http://imageJ.nih.gov/ij/index.html) and 3 to 5 cells with intact body were randomly selected by a masked investigator. The number of extensions was counted. The area of the cell body and the longest extension length were determined using Image J. We combined these measurements into a new parameter that we named ‘microglial morphology activation value’ (MMAV): MMAV = [cell body area]/[(largest extension length) × (# of extensions)].

### Immunohistochemistry of retinal sections

Since the subretinal MG/MΦ are localized precisely where artifactual retinal detachments occur during regular specimen preparation for retinal sections, we used a quick-freezing of eyeballs followed by a freeze-substitution technique. This method has been shown to be effective in 1) preventing artifactual retinal detachment, 2) minimizing ice crystal formation, 3) preserving cellular morphology, and 4) preserving antigenicity [30–34].

Eyes (n = 8 eyes per group) were enucleated and immediately placed in 2-ml tubes with holes and frozen in liquid nitrogen-cooled isopentane for 2 minutes. The tubes were immediately transferred to liquid nitrogen. After all eyes were collected, the tubes with frozen eye balls were transferred to a pre-cooled solution of methanol/acetic acid (97:3; in -80°C freezer) in 50-ml tubes for freeze substitution for at least 48 h. The eyes were gradually warmed up to room temperature (first at -20°C for 24 h, then at 4°C for 4 h, and finally moved to RT). The eyes were then transferred to 100% ethanol for paraffin embedding using routine methods.

Sections were double stained with pairs of primary antibodies (rabbit anti-Iba1 combined with either mouse anti-iNOS, or anti-MHC class II-FITC, or rat anti-CD16/32), followed by secondary antibodies (see Additional file 2: Table S2). Primary antibodies were omitted in the control sections. All samples were imaged on a Leica DMI300B Microscope equipped with a Hamamatsu ORCA flash 4.0 camera, using Leica LAS AF software at 40X magnification. The FITC channel (AF488) was used for iNOS, MHC-II and CD16, while the Texas-Red channel (AF 594) was used for Iba-1.

### Electron microscopy

After cardiac perfusion with 1% glutaraldehyde and 2% paraformaldehyde in PBS (pH 7.4), fixed eyes were removed and sectioned behind the limbus, and posterior eyecups were processed as described before [21]. In brief, eyes were post-fixed in osmium tetraoxide, dehydrated in ethanol series, and embedded in Poly/Bed 812 epoxy resin (Polysciences, Inc., Warrington, PA, USA). For electron microscopy (EM), 70-nm thin sections were cut, stained with 2% aqueous uranyl acetate and lead citrate, and imaged using a JEOL 1200EX II transmission electron microscope (JEOL USA, Inc) at the UT Southwestern Medical Center EM Core Laboratory.

### RNA extraction from retinal pigment epithelium and retina of B6-WT and rd8 mutant mice

Eyes were enucleated and the anterior segment was removed. The retina and the remaining posterior eye cup (containing the RPE cells and overlying subretinal microglia) were separated and processed differently. The retina was placed in 500 μl Qiazol lysis reagent and was then homogenized using the Bio-Gen PRO200 Homogenizer (Pro Scientific, Oxford, CT, USA). The Qiagen miRNeasy MiniKit (Qiagen Sciences, LLC, Germantown, MD, USA; cat: 217004) was then used to isolate the total RNA. In the meantime, the posterior eye cup was quickly dipped in PBS in order to quickly wash out any adherent debris and immediately transferred into a 1.5 ml microcentrifuge tube containing 200 μl of RNAprotect cell reagent (Qiagen, cat. 76526). It took roughly 1 minute from the time of enucleation to the transfer into the RNAprotect-containing microcentrifuge tube. The posterior eye cup was then processed using the SRIRS (simultaneous RPE isolation and RNA stabilization) method that we described [35] in order to isolate a high quality and quantity RNA specifically derived from the RPE cells and overlying subretinal MG/MΦ.

### Quantitative RT-PCR

We used the Superscript III reverse transcriptase kit (Invitrogen Inc., Grand Island, NY, USA; cat. 11735-032) to generate cDNA from the extracted total RNA. Singlet qPCR reactions were run in triplicate (iCycler; Bio-Rad Laboratories, Hercules, CA, USA) at 95°C for 3 minutes, followed by 40 cycles of 95°C for 15 seconds and 60°C for 1 minute with SYBR Green ER qPCR SuperMix (Invitrogen, cat. 11735-032). Each reaction contained 2.5 ng cDNA, 200 nM of each primer, and 10 μl qPCR super mix in 20 μl total volume. The primers used are shown in Additional file 3: Table S3 and Additional file 4: Table S4. The fold changes in expression of the genes in the RPE/microglia cell isolates were calculated using the formula RQ = 2^−ΔΔCt^, and using GAPDH as an endogenous reference gene.

### Statistical analysis

SigmaPlot 11.0 (Systat Software Inc., San Jose, CA, USA; http://www.sigmaplot.com) and/or Microsoft Excel was used for statistical analysis. Data are presented as the mean ± standard error of mean (SEM). A two-tailed Student’s *t*-test was performed when comparing two groups and a one-way analysis of variance (ANOVA) was applied to assess differences between groups, followed by Tukey’s Test for all pairwise multiple comparison procedures when necessary, and *P* value <0.05 was considered significant.

## Results

### The distribution of yellow fundus spots on B6-mice changes with age and rd8 mutation

**Figure 1:**
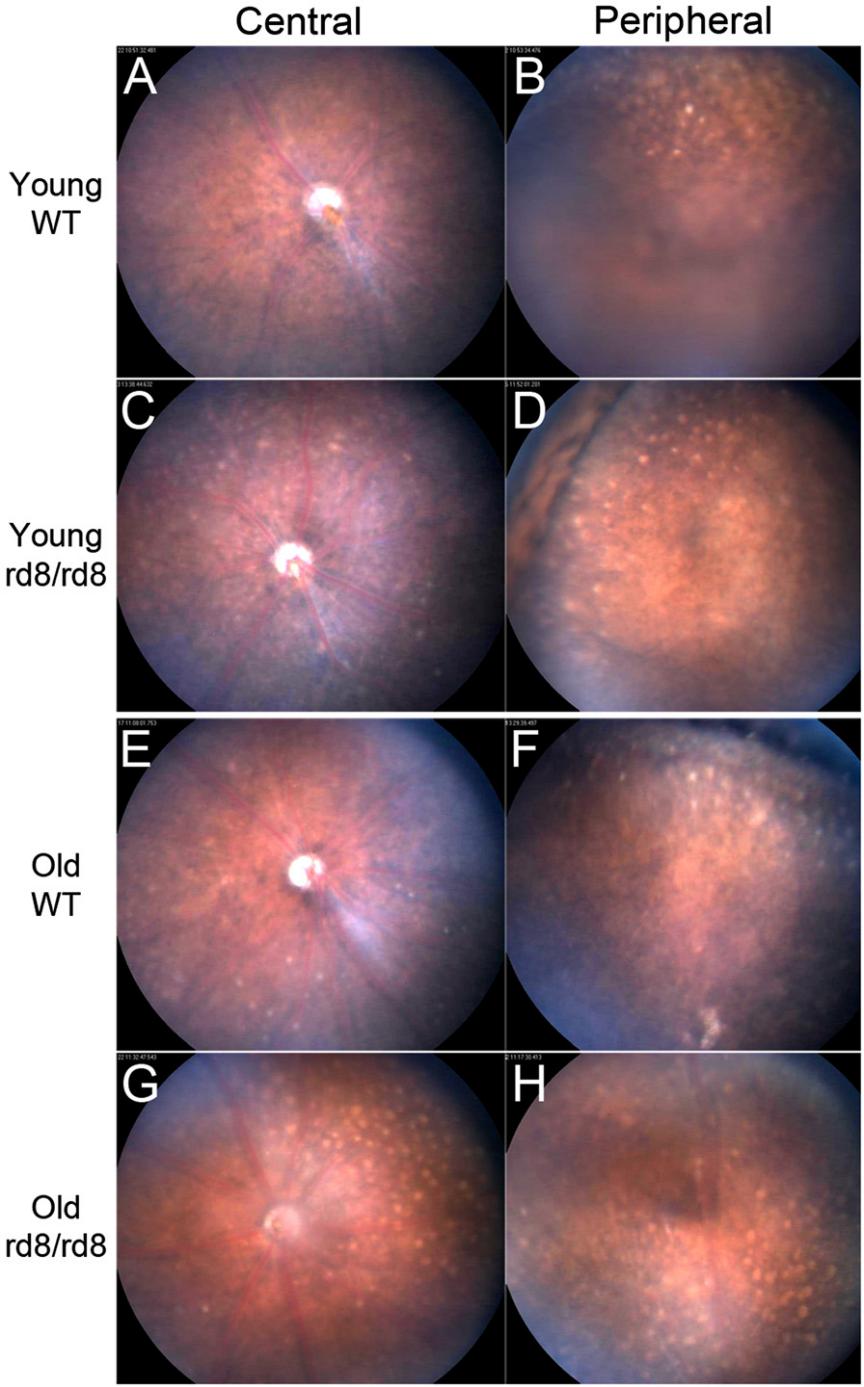
**Photographs of central and peripheral retina in C57BL/6N (rd8/rd8) and C57BL/6 J (wild-type) mice.** A change in the distribution of fundus spots on B6-mice due to both age and the presence of the rd8 mutation is seen. Yellow spots are shown in central **(A, C, E, and G)** and peripheral **(B, D, F, and H)** fundus photographs of representative young **(A-D)** and old **(E-H)** B6-mice. Mice 2 to 8 months of age were classified as ‘young’, while mice 14 to 20 months of age were classified as ‘old’. Note that the number of central fundus spots is increased in old age for both rd8 mutant (G versus C) and wild-type (WT) (E versus A) mice. Furthermore, rd8/rd8 mice show a marked increase in central spots compared to WT, both in the young (C versus A) and old (G versus E) age groups.

**Figure 2:**
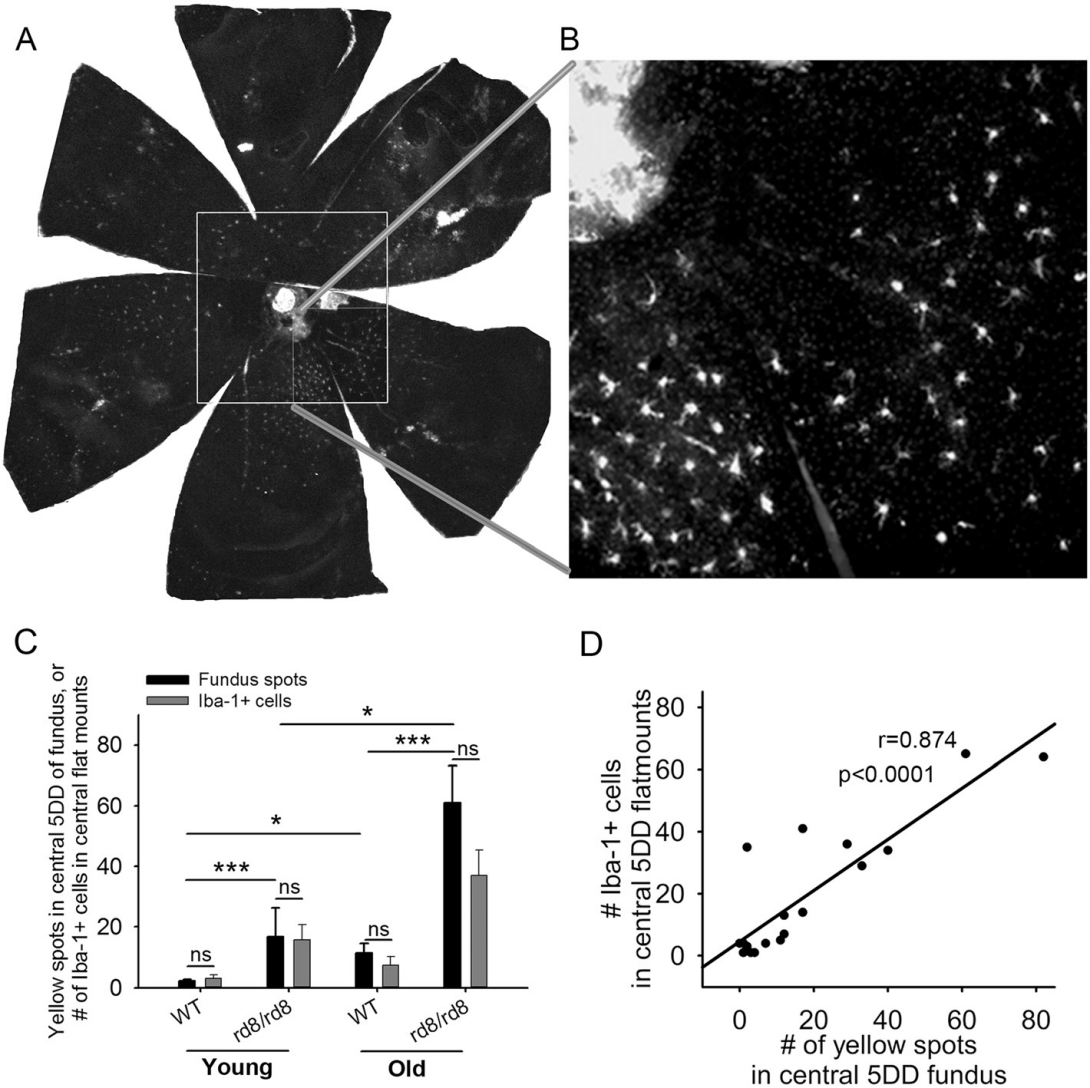
**Central fundus spots increase with age in both B6 groups, most prominently in rd8/rd8 mice. (A)** A retinal pigment epithelium (RPE) flat mount of an old rd8 mouse stained for ionized calcium binding adaptor (Iba)-1 is shown to demonstrate the area counted as the ‘central flat mount’ (large white square). The ‘central flat mount’ is made up of four higher magnification photos (magnification 10X/1.6X optivar) taken around the disc (smaller squares). **(B)** One of those higher magnification photos is shown. **(C)** The number of yellow spots (black bars) in the central fundus (within a circle with a 5 disc diameter (DD) radius and centered on the disc) is very similar to the number of Iba-1+ cells (gray bars) on the corresponding central flat mounts. There is an increase in central fundus spots in rd8/rd8 mice compared to wild-type (WT) mice in each age group. Furthermore, there is an increase in the number of central yellow spots with age in both genotypes (young WT (n = 11), young rd8 (n = 3); old WT (n = 11), old rd8 (n = 10)). **(D)** Linear regression showing a significant correlation between the number of yellow spots in central fundus (5 DD radius) and the number of Iba-1 positive cells in the corresponding flat mount area (n = 19 eyes). Mice 2 to 8 months of age were classified as ‘young’, while mice 14 to 20 months of age were classified as ‘old’. *P <0.05, ***P <0.001.

Fundus examination of C57BL/6 mice revealed yellow spots in mice of all ages (Figure 1). In young B6 mice (2- to 8 mo) of both genotypes, the vast majority of the spots were located in the far retinal periphery, close to the ora serrata (Figure 1B and D). In this age group, a small number of yellow spots were usually seen in the posterior retina of C57BL6/N mice. However, these spots were seen only rarely in the posterior retina of young C57BL/6J mice (Figure 1A and C). As mice aged, the distribution of yellow spots shifted. In old mice (14-to 20 mo), the geographic distribution of these spots shifted towards the mid-peripheral and central retina (Figure 1E,G, and black bars in Figure 2C) in both genotypes. However, this change was most accelerated in the C57BL/6N rd8 mutant mice. The number of yellow spots in the central retina was significantly higher in rd8 mutant compared to WT mice (Figure 1A versus C, E versus G, and black bars in Figure 2C) for both age groups.

### The microglia/macrophage cell count on retinal pigment epithelium flat mounts corresponds to the number of fundus yellow spots

Quantitative analysis was performed for the 1) yellow spots in the central fundus (5 DD radius from the nerve), 2) Iba-1+ cells on the RPE flat mounts within a similar distance of the disc, and 3) Iba-1+ cells on the entire RPE flat mounts. Figure 2A shows an entire flat mount stained for Iba-1, and the area that was counted as the central flat-mount (large white square). Four higher magnification photos (Figure 2B) around the nerve were taken and the Iba-1+ cells in these images were counted and reported as the ‘central flat mount’ count. There was a striking similarity in the number and distribution of yellow spots on the fundus compared to the Iba-1+ cells on the corresponding RPE flat mounts. This was true for both B6-WT and rd8 mutant mice (Figure 2C). The correlation (approaching a 1:1 ratio) between the number of yellow spots in fundus photos and the number of Iba-1+ cells in the flat mounts was strong (Figure 2D, r = 0.874, *P* <0.0001, n = 19 eyes).

**Figure 3:**
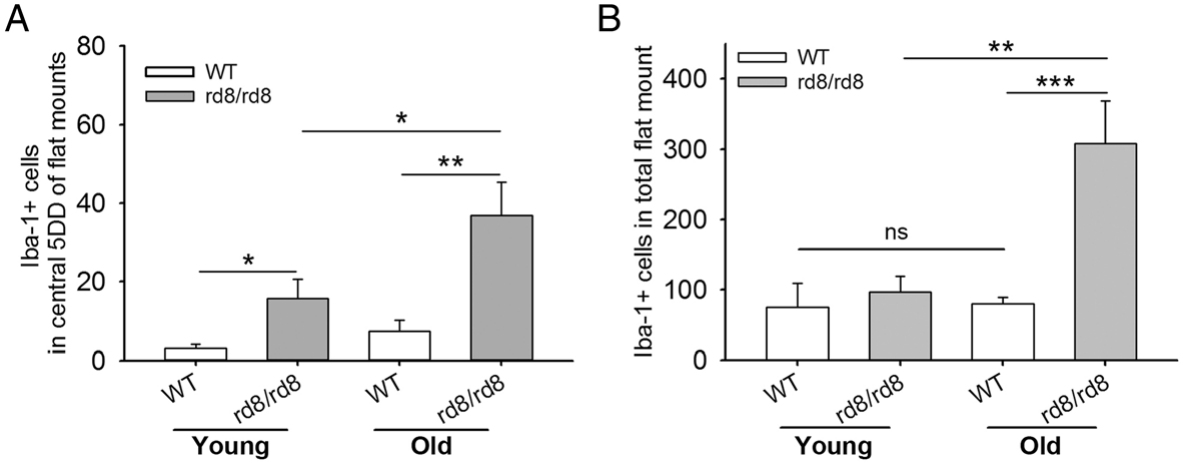
**The total number of subretinal microglia/macrophages (MG/MΦ) increases with age in C57BL/6N, but not C57BL/6J mice. (A)** Number of ionized calcium binding adaptor (Iba)-1 positive cells in the central flat mount of young and old mice showing a significant increase in both age groups of rd8/rd8 mice compared to the age-matched wild-type (WT). **(B)** Number of Iba-1 positive cells in the entire/total retinal pigment epithelium (RPE) flat mounts showing that the total number of subretinal microglia increases with age in rd8/rd8 mice, but not in WT mice. The graphs represent the combined results of two to three experiments, which included young mice (WT, n = 10 eyes; or rd8/rd8, n = 8 eyes), and old mice (WT, n = 13 eyes; or rd8/rd8, n = 11 eyes). Mice 2 to 8 months of age were classified as ‘young’, while mice 14 to 20 months of age were classified as ‘old’. *P <0.05, **P <0.01, ***P <0.001.

Similar to our findings on fundus yellow spots, the central counting of Iba-1+ cells on flat mounts was significantly higher in the rd8 mutant mice compared to WT mice. This was the case, in young mice (young rd8 > young WT, *P* <0.05) and also in old mice (old rd8 > old WT, *P* <0.005) (Figure 3A).

When we counted the total number of Iba-1+ subretinal cells, including those in the periphery, we found that rd8 mutant mice demonstrated a significant increase with age (*P* <0.01, Figure 3B). In contrast, aging did not lead to an increase in the total number of subretinal MG/MΦ in WT mice (*P* = 0.9, Figure 3B). This finding was confirmed in a subgroup analysis (3 to 6 mo, n = 9; 15 mo, n = 6; and 18 to 19 mo, n = 7) as shown in Additional file 5: Figure S1. Comparing the two genotypes by age (again, counting Iba-1+ cells in the entire flat mounts), there was no difference in the number of subretinal MG/MΦ in young rd8/rd8 mice versus young WT mice. Yet, there was a dramatic increase in the total number of Iba-1+ subretinal MG/MΦ in old rd8/rd8 mice compared to old WT mice (*P* <0.001).

### The phenotype of subretinal macrophages/microglial cells on retinal pigment epithelium flat mounts of B6-mice changes with age and with the rd8 mutation

**Figure 4:**
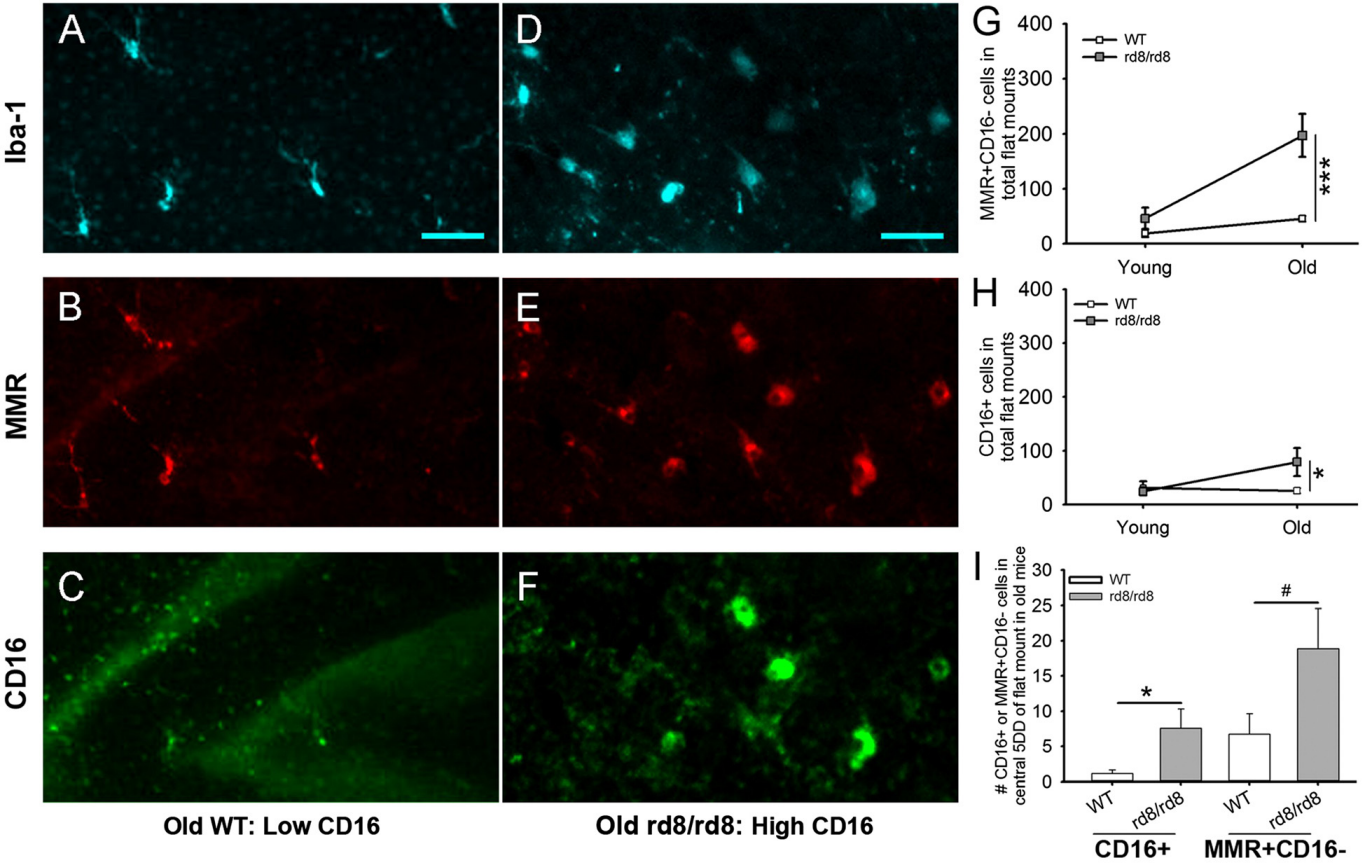
**The phenotype of subretinal microglia/macrophages in B6-mice changes with age and with the rd8 mutation. (A-F)** Representative images of triple-stain immunohistochemistry (IHC) show staining with ionized calcium binding adaptor (Iba)-1 (A and D), Macrophage mannose receptor (MMR) (B and E), and FcγIII/II Receptor (CD16/CD32, abbreviated as CD16) (C and F) of subretinal microglia/macrophages (MG/MΦ) on retinal pigment epithelium (RPE)-flat mounts. Different staining patterns are shown here: some Iba-1+ cells stain strongly for CD16 (D and F), while some Iba-1+ cells show weak or no staining for CD16 (A and C). **(G)** Quantification of MMR + CD16- MG/MΦ in the entire RPE-flat mount shows that in old mice, there is a significant increase in MMR + CD16- cells in rd8/rd8 mice compared to wild-type (WT) mice. **(H)** In the entire flat mount, there is also a significant increase in the number of CD16+ subretinal MG/MΦ in old rd8/rd8 mice compared to old WT. **(I)** The central flat mounts demonstrate a significant increase in CD16+ MG/MΦ in old rd8/rd8 mice compared to old WT mice. There is also a trend towards an increase in the number of MMR + CD16- cells in the central flat mounts of old rd8/rd8 mice compared to WT. For figures G-I we combined three similar experiments using 10 young WT, 8 young rd8/rd8, 13 old WT and 10 old rd8/rd8 eyes. Mice 2 to 8 months of age were classified as ‘young’, while mice 14 to 20 months of age were classified as ‘old’. *P <0.05, ***P <0.001, #*P* = 0.056.

Next, we aimed to determine whether the subretinal MG/MΦ expresses different markers, and whether their phenotype changes with age or in relation to the presence of the rd8 mutation. To this end, we stained RPE flat mounts for Iba-1, MMR (a scavenger receptor), and/or CD16 (a pro-inflammatory marker). Figure 4 illustrates some of the different patterns of staining seen in our experiments. We sometimes observed cells that had strong CD16 staining (Figure 4F), while in other cases the CD16 staining was very weak (Figure 4C). It was not uncommon to find cells that were simultaneously positive for Iba-1, CD16 and MMR. The phenotype of subretinal MG/MΦ of both genotypes changed with age. The proportion of Iba-1+ MG/MΦ that were also MMR + CD16- increased with age (Figure 4G) in both WT and rd8/rd8 mice. However, the increase was more pronounced in rd8 mutant mice. Thus, in the old age group there was a significant increase in MMR + CD16- cells in rd8 mutant mice compared to WT (*P* <0.001). Similarly, the number of CD16+ cells was increased in old rd8 mutant mice compared to WT (Figure 4H, *P* <0.05). Interestingly, when we looked at the central retina of old mice, there was also a statistically significant increase in CD16+ cells in rd8 mutant mice compared to WT (Figure 4I, *P* <0.05). A trend towards an increase in MMR + CD16- cells in the central retina of old rd8 mutant mice was also seen, but did not reach statistical significance (*P* = 0.056).

### The CD16+ subpopulation of subretinal microglia/macrophages in both, old B6-WT mice, and particularly rd8 mutant mice, show morphological signs of activation

**Figure 5:**
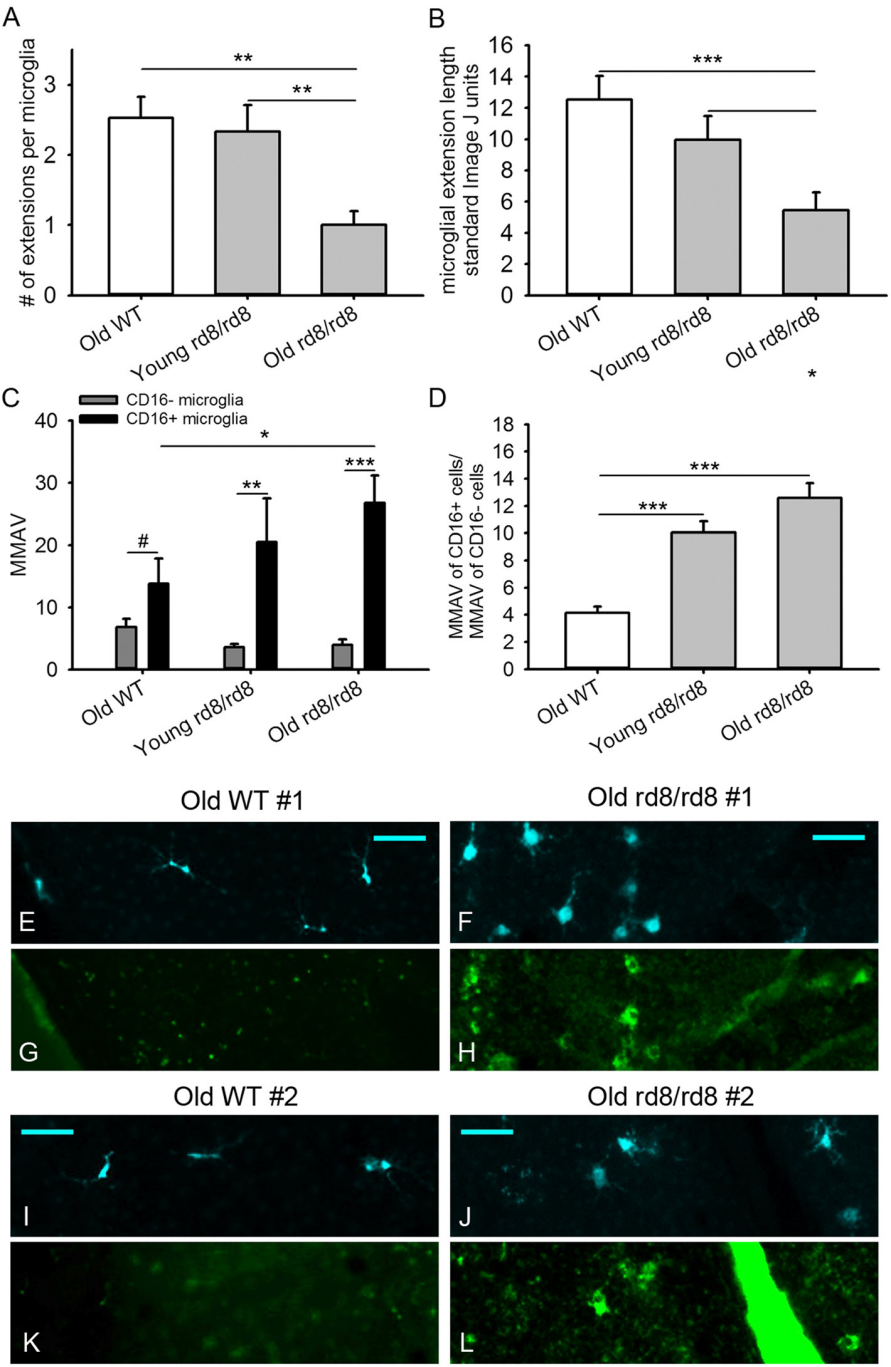
**Morphological analysis of subretinal microglia/macrophages in B6-mice.** There is an increased microglia/macrophages (MG/MΦ) activation morphology in the rd8/rd8 mice, which is accentuated in old age. **(A)** The average number of extensions per MG/MΦ cell is decreased in old rd8/rd8 mice compared to both old wild-type (WT) mice and young rd8/rd8 mice. **(B)** The average length of the MG/MΦ cell extensions (measured using imageJ, http://imageJ.nih.gov/ij/index.html, and expressed as standard arbitrary units) of old rd8/rd8 mice is decreased relative to both old WT mice and young rd8/rd8 mice. **(C)** Quantification of MG/MΦ activation using the new parameter, microglial morphology activation value (MMAV) is shown. MMAV combines several morphological changes known to be associated with MG/MΦ activation into a single value, and is defined as the area of the MG/MΦ cell body divided by the product of the number of extensions and the average extension length. MMAV is increased in FcγIII/II Receptor (CD16/CD32, abbreviated as CD16) positive cells, particularly in old rd8 mutant mice. **(D)**. The ratio of MMAV for CD16+ to MMAV for CD16- cells is markedly increased in both young and old rd8 mutant mice compared to old WT mice. Two similar experiments were combined (see methods; n = 3 to 5 eyes per group, and 3 to 5 photographic fields per eye, containing 3 to 5 cells with intact cell body per field, which were randomly selected by a masked investigator). Examples of the ionized calcium binding adaptor (Iba)-1 **(E,F,I,J)** and CD16 **(G,H,K,L)** staining of MG/MΦ in two C57BL/6J (E,G,I,K) versus two C57BL/6N mice (F,H,J,L) are shown. Mice 2 to 8 months of age were classified as ‘young’, while mice 14 to 20 months of age were classified as ‘old’. *P <0.05, **P <0.01, ***P <0.001, #*P* = 0.051.

Having seen that aging and the presence of the rd8 mutation cause phenotypic changes in the subretinal MG/MΦ of B6 mice, we wondered if these changes were accompanied by morphological signs of activation [36]. We confirmed that injection of lipopolysaccharides (LPS) intraperitoneally (i.p.) into WT mice led to a marked increase in the accumulation of subretinal MG/MΦ and that those cells demonstrated the typical morphologic changes associated with activation, including larger cell bodies, shorter extensions and a lower number of extensions (see Additional file 6: Figure S2). Thus, we decided to quantitatively analyze the morphological activation of subretinal MG/MΦ on the RPE flat mounts of rd8 mutant and B6-WT mice, by measuring the cell body area and the extension length using ImageJ software. We also counted the total number of extensions per cell and derived a single parameter, ‘microglial morphology activation value’ (MMAV): MMAV = (cell body area)/((largest extension length) × (# of extensions)). Subretinal MG/MΦ in B6 mice of both genotypes were divided into CD16+ and CD16- subgroups and separately analyzed using the MMAV. The CD16+ cells of old rd8 mutant mice showed a significant decrease in the number of extensions per cell, and the extension length (Figure 5A and B) when compared to both old WT and young rd8 mutant mice. There were not enough CD16+ cells in the central young B6-WT mice to include in the analyses. There were no differences in these measurements for CD16- cells among the groups (data not shown). Within each group of mice, we found that the CD16+ subpopulation of Iba-1+ cells had a significantly higher MMAV compared to the CD16- subpopulation of Iba-1+ cells. Furthermore, the magnitude and significance of the difference increased with age, and with the presence of the rd8 mutation (Figure 5C). When the difference in the activation morphology of CD16+ versus CD16- cells for each group of mice was quantified, by calculating the ratio (MMAV for CD16+)/(MMAV for CD16-), it was three times higher in old rd8 mutant mice compared to old WT mice (*P* <1 × 10^−8^, Figure 5D). Examples of the Iba-1 and CD16 staining of MG/MΦ cells in two C57BL/6J mice (Figure 5E,G,I,K) versus two C57BL/6N mice (Figure 5F,H,J,L) are shown.

### Subretinal microglia/macrophages in C57BL/6N mice demonstrate other signs of activation

**Figure 6:**
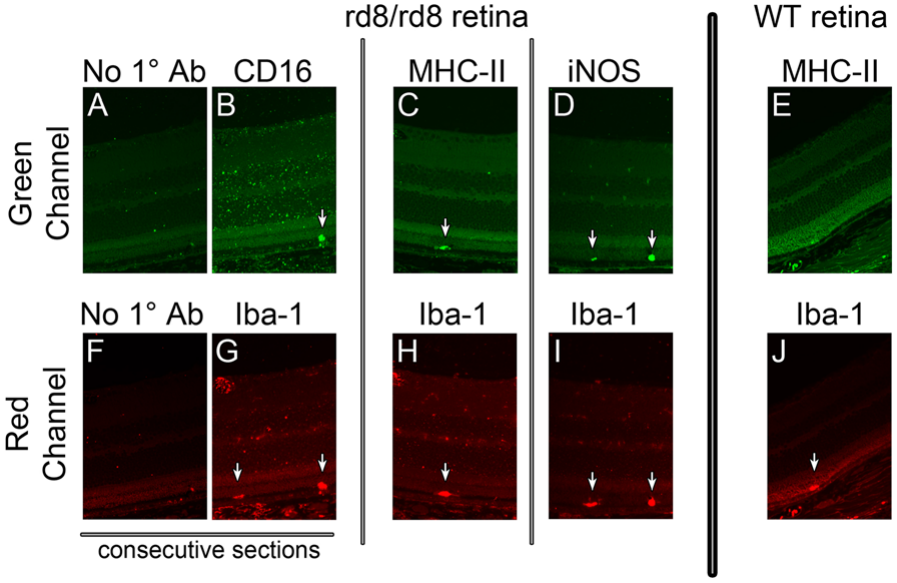
**Subretinal microglia/macrophages (MG/MΦ) in C57BL/6N mice express markers of activation.** After processing eyes using the freeze-substitution technique (see Methods, n = 8 eyes per group), retina sections from C57BL/6N **(A-D and F-I)** or from C57BL/6J **(E and J)** eyes were obtained and stained. Each section was double-labeled with ionized calcium binding adaptor (Iba)-1 (red channel; G-J) and one microglia/macrophage activation marker (green channel; B-E). A control sample is also shown (secondary antibodies without primary antibodies; A and F), which is a consecutive section of the one stained with Iba-1 and FcγIII/II Receptor (CD16/CD32, abbreviated as CD16) (B and G). Note the expression of CD16 and activation markers MHC-II and iNOS in subretinal MG/MΦ in rd8 mutant samples (arrows in B,C,D). There is no staining for MHC-II in the wild-type (WT) subretinal MG/MΦ (E versus C).

In order to corroborate that the subretinal MG/MΦ in rd8/rd8 C57BL/6N mice express markers of activation, we obtained retina sections using a freeze-substitution technique and performed IHC for Iba-1 in combination with CD16, MHC-II or iNOS. We confirmed that Iba-1+ MG/MΦ localize to the interface between RPE and photoreceptors and that some of them express CD16 (Figure 6B). More importantly, we found that Iba-1+ subretinal cells demonstrated expression of MHC-II and iNOS in C57BL/6N (Figure 6 C,D) but not in WT eyes (Figure 6E).

**Figure 7:**
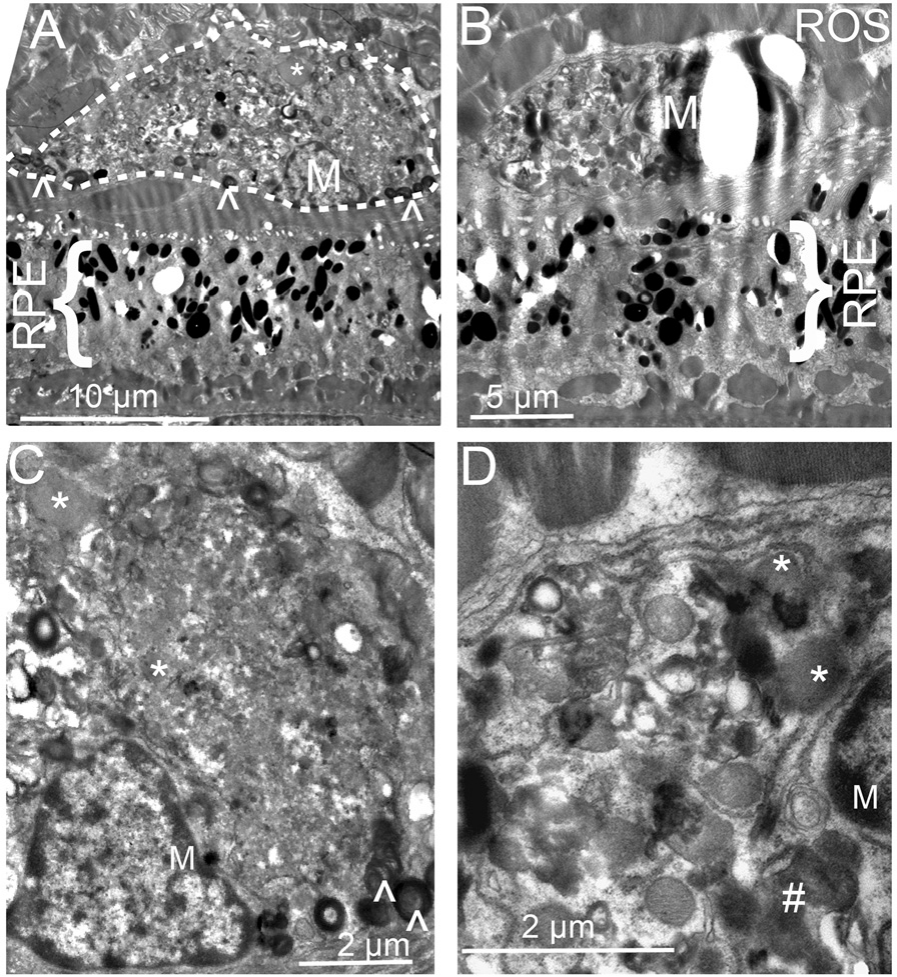
**Electron microscopy of retinal sections in old rd8/rd8 mice.** Cells consistent with microglia/macrophages (MG/MΦ) were seen between the retinal pigment epithelium (RPE) and the photoreceptor outer segments **(A,B)**. Higher magnification images from these cells **(C,D)** demonstrate the presence of photoreceptor outer segment debris (labeled with ^), occasional melanosomes, lipofuscin granules (*), and multiple phagosomes with partially degraded debris (#). The photoreceptor outer segments seen over the microglia in A and B are labeled as ROS (rod outer segments).

Finally, EM of retinal sections in old rd8/rd8 mice revealed the presence of cells between the RPE and the photoreceptor outer segments (Figure 7A,B). The cytoplasm of these cells was full of ingested whorls of photoreceptor debris (labeled with ^), lipofuscin granules (*), multiple phagosomes with partially degraded debris (#), and occasional melanosomes. These are findings consistent with actively phagocytic MG/MΦ (Figure 7A-D).

### Inflammatory and oxidative stress genes are differentially expressed in the retinal pigment epithelium and retina of rd8 mutant mice compared to wild-type mice

**Figure 8:**
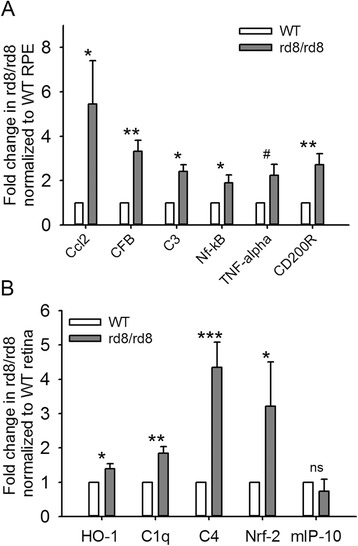
**RT-qPCR analysis of RNA from retinal pigment epithelium (RPE)/subretinal microglia/macrophages (MG/MΦ) isolates and from retina isolates.** There is an upregulation of genes related to oxidative stress, complement activation and inflammation in rd8 mutant mice compared to wild-type (WT). **(A)** Fold changes in the expression of target genes, in RNA isolated from RPE/subretinal MG/MΦ from rd8 mutant mice, normalized to B6-WT. The bars represent the average of two experiments in age-matched 6- to 8-month-old mice, including a total of 10 rd8/rd8 and 10 WT eyes, except for *CFB* (6 rd8/rd8 and 6 WT eyes). **(B)** Fold changes in the expression of target genes in RNA isolated from the retina of rd8 mutant mice normalized to B6-WT. The bars represent the average of two experiments including a total of 11 rd8/rd8 and 11 B6 eyes. *P <0.05, **P <0.01, ***P <0.001, #*P* = 0.058.

Analysis of gene expression by RT-qPCR showed the differential expression of several genes related to oxidative stress, complement activation, inflammation, and MG/MΦ chemoattraction, in the RPE and retina of rd8/rd8 mice compared to wt/wt mice (Figure 8). In the RNA isolates from the RPE/subretinal MG/MΦ of rd8 mutant mice the following six genes were upregulated compared to the B6-WT controls (Figure 8A): *Ccl2* (5.5-fold, *P* <0.05), *CFB* (3.3-fold, *P* <0.005), *C3* (2.4-fold, *P* <0.05), *NF-kβ* (1.9-fold, *P* <0.05), *CD200R* (2.7-fold, *P* <0.005) and *TNF-alpha* (2.2-fold, *P* = 0.058). We suspected that these pro-inflammatory genes were increased due to oxidative stress and complement activation in the retina of rd8 mutant mice. This hypothesis was supported by our findings of increased expression of oxidative stress, complement activation and inflammation related genes in the retina of rd8 mutant mice (Figure 8B): *HO-1* (1.4-fold, *P* <0.05), *C1q* (1.8-fold, *P* <0.01), *C4* (4.3-fold, *P* <0.001), and *Nrf-2* (3.2-fold, *P* <0.05).

## Discussion

### The geographic distribution of subretinal microglia/macrophages changes with age in both C57BL/6N and C57BL/6J mice, but the total number of microglia only increases in C57BL6/N mice

In this work, we describe the clinical appearance and natural history of subretinal MG/MΦ in naïve C57BL/6 mice with (C57BL/6N or ‘rd8 mutant’), or without (C57BL/6J or WT) the rd8 mutation of the *Crb1* gene. The strong correlation between yellow fundus spots and subretinal Iba-1+ cells suggests that most of the yellow drusen-like fundus spots we observed are in fact subretinal MG/MΦ. This is in line with observations by several groups [13,19,37]. Of course, this does not rule out the possibility that a relatively small number of these spots could represent intra-retinal lesions in the rd8 mutant mice, as some groups have suggested [25,38]. We also found that in C57BL/6J mice (in the absence of the rd8 mutation), the total number of subretinal MG/MΦ appears to remain stable with age. On the other hand, we observed that aging does lead to an increase in the total number of subretinal MG/MΦ in C57BL/6N mice. A large portion of the subretinal MG/MΦ in young mice are located in the far retinal periphery and may be missed by regular fundus photos or common methods of flat mount preparation.

Our data shows that as mice age, the distribution of the yellow fundus spots (and the corresponding Iba-1+ cells) changes: they seem to localize further away from the ora serrata and more towards the central retina. These changes developed earlier and more prominently in the rd8 mutant mice. In the rd8 mutant mice, there was also some predilection for the inferonasal quadrant, but we often found spots throughout the entire posterior fundus. We have not determined if the ‘centripetal’ change in distribution of fundus spots is due to migration of cells within the subretinal space, or if the more posterior cells are originating from subsequent waves of microglial migration from the inner retina and into the subretinal space in a different area of the fundus. Experiments by two groups [12,39] showed that subretinal microglia completely turned over in 2 to 6 months. Assuming that their observations apply to naïve mice, the distribution of subretinal microglia at any given time may be determined by chemoattractants secreted by RPE cells in response to factors related to aging or retinal pathology (such as the retinal degeneration associated to the rd8 mutation).

### With aging, an increasing proportion of subretinal microglia/macrophages in the C57BL/6N (rd8 mutant) mice demonstrate a pro-inflamatory phenotype and activated morphology compared to C57BL/6J

In studying phenotype, we used CD16 as a marker for pro-inflammatory MG/MΦ [40–42]. CD16+ cells have been shown to have a higher expression of proinflammatory cytokines and higher potency in antigen presentation [43]. It has been used as a marker of activated MG/MΦ both in humans [40,43–45] and in mice [41,42,45–51]. We used MMR (CD206 or MRC-1) as a marker for a scavenging phenotype [52,53]. MMR may have a role in promoting clearance and reduced immunogenicity of glycoproteins [54]. Some groups have also used CD16 as a marker of M1 macrophage phenotype [40–42], and MMR as a marker of M2 phenotype [41,42,54]. Yet, macrophages do not always conform to a M1 versus M2 classification [55,56]. In fact, in our studies we observed that it is not uncommon for MG/MΦ to express both MMR and CD16 simultaneously.

As the WT mice aged, they had an increase in MMR + CD16- cells, while CD16+ cells remained stable or slightly decreased. This suggests a change towards a scavenging phenotype. However, in the old rd8 mutant mice, both the MMR + CD16- and the CD16+ cells were increased relative to the WT mice. Importantly, we found robust morphologic changes of activation in the CD16+ subpopulation of subretinal MG/MΦ, and these were most prominent in the aging rd8 mutant mice. Old rd8 mutant mice also had ultrastructural (EM) and immunohistochemical evidence of activated MG/MΦ between the RPE and the photoreceptor outer segments. Subretinal MG/MΦ in rd8 mutant mice demonstrated expression of the activation markers iNOS and MHC-II. These findings are interesting in view of recent observations by Murinello *et al*. [46] showing an increase in CD16 expression (accompanied by increased MHC-II expression) in retinal MG/MΦ in an acute inflammatory model, and increased expression of FcγRII (one of the antigens recognized by our CD16/CD32 antibody) in MG/MΦ of early and late AMD retinal and choroidal specimens.

Some studies suggest that MG/MΦ play a protective role in AMD (by clearing pro-inflammatory debris) [11,57–59], while others suggest that they promote disease progression (perhaps by secreting pro-inflammatory or pro-angiogenic factors) [59–61]. This dichotomy has also been described by others in the setting of uveal melanoma [62], and Alzheimer’s disease [2,3]. It is thought that as mice age, there is an increase in subretinal debris and basal laminar deposits, even in naïve mice [63,64]. We propose that in normal aging and in the absence of retinal pathology (as in B6 mice that do not have the rd8 mutation), the MG/MΦ are accumulating posteriorly and changing towards a more defined scavenger phenotype in order to help handle this accumulation of debris. The fact that autofluorescent lipofuscin accumulates in subretinal MG/MΦ with age [65] suggests that one of their roles is to clear waste products from the normal turnover of photoreceptors. As Wong’s group and others have suggested [36], this may involve a form of activation. However, our data suggest that this normal activation is quantitatively (low MMAV), and qualitatively (MMR + CD16-) different from the activation seen in the diseased retina of rd8 mutant mice. When aging is accompanied by retinal pathology (as in the rd8 mutant mice), among the subretinal MG/MΦ there is an increased number of cells characterized by a more pro-inflammatory (CD16+) phenotype. Finally, our finding of markedly different MMAVs for CD16+ MG/MΦ in rd8 mutant versus WT mice suggests that MG/MΦ activation in the subretinal space is a gradated process, not an all-or-none phenomenon.

### Oxidative stress, complement activation, and inflammation related genes are upregulated in the rd8 mutant mice

We found that in the rd8 mutant mice, retinal pathology is accompanied by an up-regulation of genes related to oxidative stress (*HO-1* and *Nrf-2*), and complement activation (*C1q* and *C4*) in retina extracts compared to WT mice. Interestingly, a recent study [46] demonstrated deposition of *C1q* in early AMD specimens. Simultaneously, or perhaps as a consequence, the RPE of rd8 mutant mice demonstrates an upregulation of a gene involved in the chemoattraction of MG/MΦ (*Ccl-2*). The RPE/MG/MΦ cells in the rd8 mutant mice also have an increased expression of genes related to complement activation (*C3* and *CFB*), and genes related to inflammation (*TNFα*, *NF-kβ* and *CD200R*). Our findings in rd8 mutant mice of a pro-inflammatory RPE gene expression profile in association with increased MG/MΦ cell activation parameters are more interesting when we consider the results by Luo *et al*. [66]. They showed a markedly increased expression of inflammatory cytokines (iNOS and Ccl2), and complement genes (*C3* and *CFB*) by RPE cells that had been cultured with supernatants of activated macrophages. Also, Ma *et al*. [67] recently found an increase in *C3* and *CFB* in MG/MΦ isolated ex-vivo from the retinas of old C57BL/6J mice (compared to young mice). They propose that aging leads to an increase in complement activation in the retina of wild-type mice. Our findings suggest that this increase is even more pronounced (and occurs at a younger age) in mice expressing the rd8 mutation. The combination of all of these observations suggests a cross-talk between retina, RPE cells and subretinal MG/MΦ: retinal pathology leading to oxidative stress (like the rd8 mutation in the setting of still unidentified permissive genes) will lead to complement activation, and activation of RPE cells, which secrete chemoattractants for MG/MΦ. The ensuing accumulation of activated subretinal MG/MΦ may further induce the secretion of pro-inflammatory molecules by the RPE, promoting a vicious cycle.

It should be noted, however, that among the markers of oxidative stress and inflammation that we detected, several molecules have actually been proposed to have a role in controlling oxidative stress and inflammation. *Nrf-2* is usually upregulated in response to oxidative stress; but it is in fact in charge of activating the primary cellular defense mechanism against the cytotoxic effects of oxidative stress. Handa’s group has recently shown that *Nrf-2* modulates smoke-induced complement activation in the RPE [68]. Also, *CD200R* is a receptor expressed on MG/MΦ, which may be involved in inducing regulation or quiescence of MG/MΦ [69]. Thus, even in a distressed retina that is causing upregulation of inflammatory mediators, there are mechanisms in place to promote a new steady state that avoids excessive inflammation.

### Permissive/modulatory genes may be needed in addition to the rd8 mutation for full expression of the phenotype seen in C57BL/6N mice

It is important to emphasize that the ocular phenotype induced by the rd8 mutation of the *Crb1* gene appears to be modulated by other genes. For example, Luhmann *et al*. [24] showed that the absence of *Cx3cr1* gene expression increased the phenotypic changes seen in rd8 mutant mice. The same group has recently identified a region in chromosome 15 that may contain such modulatory gene(s) [25]. Furthermore, Mehalow *et al*. [22] showed that rd8/rd8 mutant mice had a wide range of phenotypic changes depending on the genetic background. In fact, it was possible to obtain rd8/rd8 mice in which the only phenotypic expression found was a disruption of the external limiting membrane of the retina (no significant increase in fundus spots, and no retinal folds or pseudorosettes in retinal histological sections) [22]. Our observations lead us to speculate that different chemoattractant/inflammatory mediator milieus can lead to accumulation of MG/MΦ that may vary in their level and type of activation, and the expression of regulatory genes. In other words, it is possible that genes that regulate MG/MΦ chemotaxis and activation may be part of the still unidentified modulatory genes involved in regulating the phenotypic expression of the rd8 mutation. There is an acute need for further work directed at identifying these modulatory genes.

## Conclusions

Our results demonstrate that C57BL/6N (Crb1^rd8/rd8^) mice have increased accumulation of subretinal MG/MΦ displaying phenotypical (CD16+), morphological, and gene-expression characteristics consistent with a pro-inflammatory shift. This is in contrast to a more scavenging, and less activated MG/MΦ phenotype in C57BL/6J mice. These findings are likely triggered by a ‘diseased retinal state’ caused by the combination of the rd8 mutation and yet unidentified permissive/modulatory genes in the C57BL/6N mice. We suspect that in the pathological settings of AMD, retinal dystrophies and human diseases involving different *CRB1* mutations (for example, Lebers congenital amaurosis, and childhood-onset dystrophies), the level of activation of subretinal MG/MΦ increases significantly. Further studies are needed to better understand MG/MΦ migration, activation and differentiation under physiologic versus pathologic conditions, with the goal of finding ways to manipulate these processes for therapeutic purposes.

## Additional files

Additional file 1: Table S1. List of antibodies and dilutions used for retinal pigment epithelium (RPE)-flatmount immunohistochemistry. Additional file 2: Table S2. List of antibodies and dilutions used for immunostaining of microglia/macrophages in mouse retina sections. Additional file 3: Table S3. Primers used for qPCR analysis of gene expression by RPE/microglia/macrophages RNA isolates. Additional file 4: Table S4. Primers used for qPCR analysis of gene expression by retina RNA isolates. Additional file 5: Figure S1. Subgroup analysis of C57BL/6J (wild-type, WT) eyes, looking at three narrow age groups, confirmed that there was no difference in the total number of subretinal ionized calcium binding adaptor (lba)-1 microglia/macrophages as WT mice aged. (n.s., no statistically significant difference). Additional file 6: Figure S2. Intraperitoneal lipopolysaccharides leads to activated subretinal microglia/macrophages. Eyes were enucleated from old B6-wild-type (WT) mice, either naive (top panels) or 16 h after intraperitoneal (i.p.) injection of 100 μl lipopolysaccharides (LPS) (bottom panels; lipopolysaccharide was from salmonella enteric serotype typhimurium, 1mg/ml, Sigma-Aldrich, Inc., St. Louis, MO, USA; Cat. No. L6143). Retinal pigment epithelium (RPE) flat mounts were prepared and stained for ionized calcium binding adaptor (lba)-1 (left panels) or Fcγ III/II receptor (CD16/CD32, abbreviated as CD16, right panels). LPS eyes had a dramatic increase in the number of subretinal microglia/macrophages (MG/MΦ) (bottom panels versus top panels). The LPS-activated MG/MΦ had large cell bodies and few extensions. Furthermore, while only a few MG/MΦ were strongly positive for CD16 in the naive mice (right top panel), many were strongly positive for CD16 in the LPS-activated mice (right bottom panel). Scale bar in top left panel.

## Abbreviations

AMD: age-related macular degeneration
ANOVA: analysis of variance
CD16/CD32: Fcγ III/II receptor
CNS: central nervous system
DD: disc diameter
EM: electron microscopy
IHC: immunohistochemistry
IACUC: institutional animal care and use committee
ip: intraperitoneal
Iba-1: ionized calcium binding adaptor -1
LPS: lipopolysaccharides
MG/MΦ: microglia/macrophages
MMAV: microglial morphology activation value
MMR: macrophage mannose receptor
NEI: national eye institute
NIH: national institute of health
PBS: phosphate buffered saline
ROS: rod outer segments
RPE: retinal pigment epithelium
RT: room temperature
RT-qPCR: reverse-transcription quantitative PCR
SEM: standard error of mean
WT (wt): wild-type.
