# Supplementary material for: Differences in the distribution, phenotype and gene expression of subretinal microglia/macrophages in C57BL/6N (Crb1rd8/rd8) versus C57BL6/J (Crb1wt/wt) mice
Source: J Neuroinflammation. 2015 Jan 15;12:6. doi: 10.1186/s12974-014-0221-4 (PMC4305240; doi:10.1186/s12974-014-0221-4)
Supplement: Additional file 1: Table S1. — List of antibodies and dilutions used for retinal pigment epithelium (RPE)-flatmount immunohistochemistry. [file 12974_2014_221_MOESM1_ESM.docx]

**Additional file 1: Table S1. List of antibodies and dilutions used for RPE-flatmount immunohistochemistry.**

| **Primary Antibody** | **Company**  **(Cat. #)** | **Dilution** | **Secondary**  **Antibody** | **Company**  **(Cat. #)** | **Dilution** |
| --- | --- | --- | --- | --- | --- |
| Rabbit Anti-Iba-1 | Wako, Inc  (019-19741) | 1:500 | AF 594 Donkey  anti-Rabbit or | Invitrogen  (A-21207) | 1:200 |
|  |  |  | CF 750 Donkey  anti-Rabbit | Biotium  (20298) | 1:200 |
| Goat Anti-MMR | R&D Systems (AF2535) | 1:50 | AF 488 Donkey  anti-Goat or | Invitrogen  (A-11055) | 1:200 |
|  |  |  | AF 594 Donkey  anti-Goat | Invitrogen  (A-11058) | 1:200 |
| Rat anti-CD16/32 | BD-Pharmingen (553142) | 1:25 | AF 488 Donkey  anti-Rat | Invitrogen  (A-21208) | 1:200 |
