# Supplementary material for: Differences in the distribution, phenotype and gene expression of subretinal microglia/macrophages in C57BL/6N (Crb1rd8/rd8) versus C57BL6/J (Crb1wt/wt) mice
Source: J Neuroinflammation. 2015 Jan 15;12:6. doi: 10.1186/s12974-014-0221-4 (PMC4305240; doi:10.1186/s12974-014-0221-4)
Supplement: Additional file 2: Table S2. — List of antibodies and dilutions used for immunostaining of microglia/macrophages in mouse retina sections. [file 12974_2014_221_MOESM2_ESM.docx]

**Additional file 2: Table S2. List of antibodies and dilutions used for immunostaining of microglia/macrophages in mouse retina sections.**

| **Primary Antibody** | **Company**  **(Cat. #)** | **Dilution** | **Secondary**  **Antibody** | **Company**  **(Cat. #)** | **Dilution** |
| --- | --- | --- | --- | --- | --- |
| Rabbit Anti-Iba-1 | Wako, Inc  (019-19741) | 1:100 | AF 594 Goat  anti-Rabbit | Invitrogen  (A-11012) | 1:200 |
|  |  |  |  |  |  |
| Mouse Anti-Nos2, IgG1 | Santa cruz  (sc-7271) | 1:50 | AF 488 Goat anti-mouse IgG1 | Invitrogen  (A-21121) | 1:200 |
|  |  |  |  |  |  |
| Mouse class II (IA-b)-FITC | GeneTex (GTX75073) | 1:25 | N/A | N/A | N/A |
| Rat anti-CD16/32 | BD-Pharmingen (553142) | 1:25 | AF 488 Goat  anti-Rat | Invitrogen  (A-11006) | 1:200 |
