# Supplementary material for: Differences in the distribution, phenotype and gene expression of subretinal microglia/macrophages in C57BL/6N (Crb1rd8/rd8) versus C57BL6/J (Crb1wt/wt) mice
Source: J Neuroinflammation. 2015 Jan 15;12:6. doi: 10.1186/s12974-014-0221-4 (PMC4305240; doi:10.1186/s12974-014-0221-4)
Supplement: Additional file 3: Table S3. — Primers used for qPCR analysis of gene expression by RPE/microglia/macrophages RNA isolates. [file 12974_2014_221_MOESM3_ESM.docx]

**Additional file 3: Table S3. Primers used for qPCR analysis of gene expression by RPE/microglia RNA isolates.**

| **Gene** | **Forward Primer** | **Reverse Primer** | **bp** | **Tm** |
| --- | --- | --- | --- | --- |
| Ccl2 | TTAAGGCATCACAGTCCGAG | TGAATGTGAAGTTGACCCGT | 129 | 60 |
| CFB | CTCGAACCTGCAGATCCAC | TCAAAGTCCTGCGGTCGT | 112 | 60 |
| C3 | AGCAGGTCATCAAGTCAGGC | GATGTAGCTGGTGTTGGGCT | 167 | 60 |
| NF-kB | AACCTGGGAATACTTCATGTGACTAA | GCACCAGAAGTCCAGGATTATAGC | 103 | 62 |
| CD200R | CAGAGAAGAGCAATCCAC | CGACAAAGTAAGGCAGTC | 98 | 55 |
| TNF | TCAGCCGATTTGCTATCTCA | CGGACTCCGCAAAGTCTAAG | 204 | 60 |
