# Supplementary material for: Differences in the distribution, phenotype and gene expression of subretinal microglia/macrophages in C57BL/6N (Crb1rd8/rd8) versus C57BL6/J (Crb1wt/wt) mice
Source: J Neuroinflammation. 2015 Jan 15;12:6. doi: 10.1186/s12974-014-0221-4 (PMC4305240; doi:10.1186/s12974-014-0221-4)
Supplement: Additional file 4: Table S4. — Primers used for qPCR analysis of gene expression by retina RNA isolates. [file 12974_2014_221_MOESM4_ESM.docx]

**Additional file 4: Table S4. Primers used for qPCR analysis of gene expression by retina RNA isolates.**

| **Gene** | **Forward Primer** | **Reverse Primer** | **Bp** | **Tm** |
| --- | --- | --- | --- | --- |
| HO-1 | TCAGGTGTCCAGAGAAGGCTTT | CTCTTCCAGGGCCGTGTAGA | 70 | 62 |
| C1q | TAGAAGCATCACAGAACA | TAGAAGCAGCAGTAACAG | 108 | 55 |
| C4 | ACCCCCTAAATAACCTGG | CCTCATGTATCCTTTTTGGA | 320 | 58 |
| Nrf-2 | CTTGCTCTTGGGAACAAGGAAC | CGACAGAAACCTCCATCTTCTG | 157 | 61 |
| IP-10 | GCTGCCGTCATTTTCTGC | TCTCACTGGCCCGTCATC | 111 | 56 |
