# Supplementary figures and images for: Differences in the distribution, phenotype and gene expression of subretinal microglia/macrophages in C57BL/6N (Crb1rd8/rd8) versus C57BL6/J (Crb1wt/wt) mice
Source: J Neuroinflammation. 2015 Jan 15;12:6. doi: 10.1186/s12974-014-0221-4 (PMC4305240; doi:10.1186/s12974-014-0221-4)

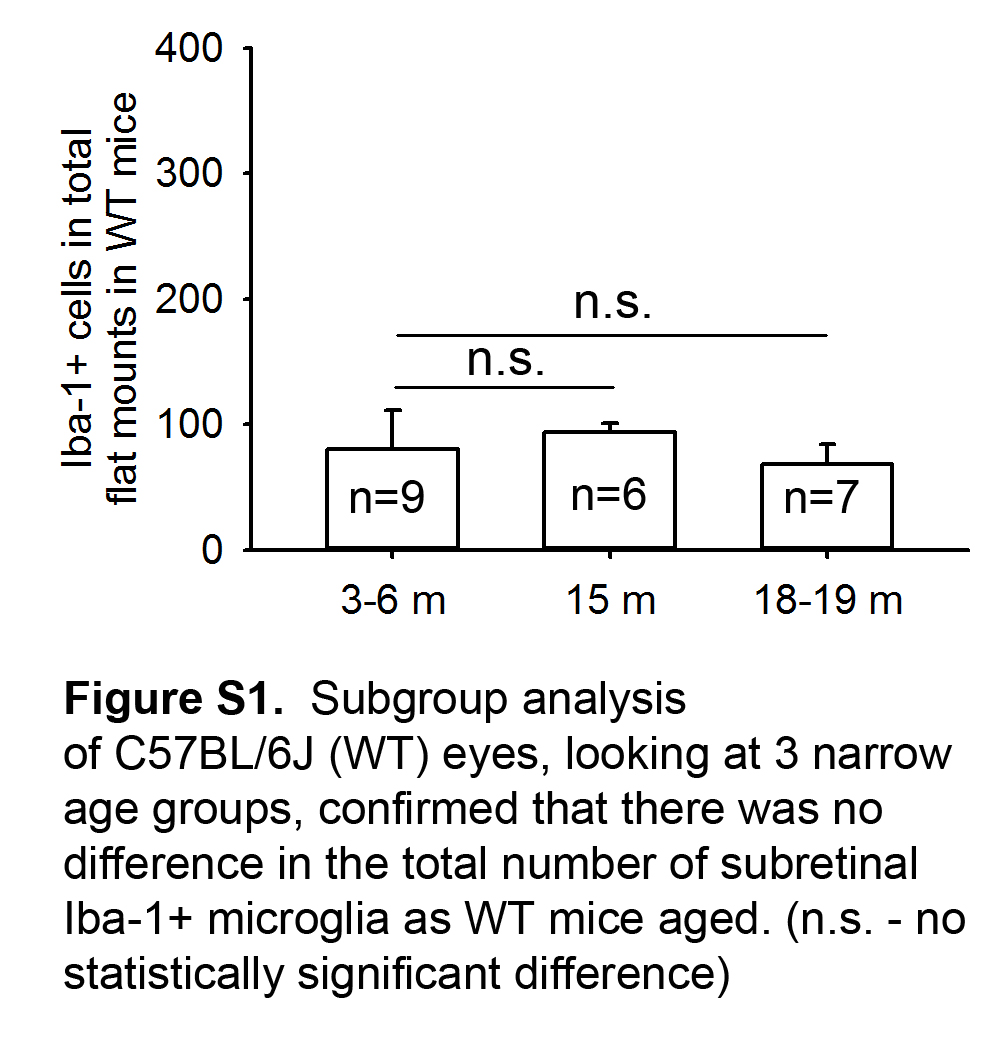

Supplement: Additional file 5: Figure S1. — Subgroup analysis of C57BL/6J (wild-type, WT) eyes, looking at three narrow age groups, confirmed that there was no difference in the total number of subretinal ionized calcium binding adaptor (lba)-1 microglia/macrophages as WT mice aged. (n.s., no statistically significant difference). [file 12974_2014_221_MOESM5_ESM.jpeg]

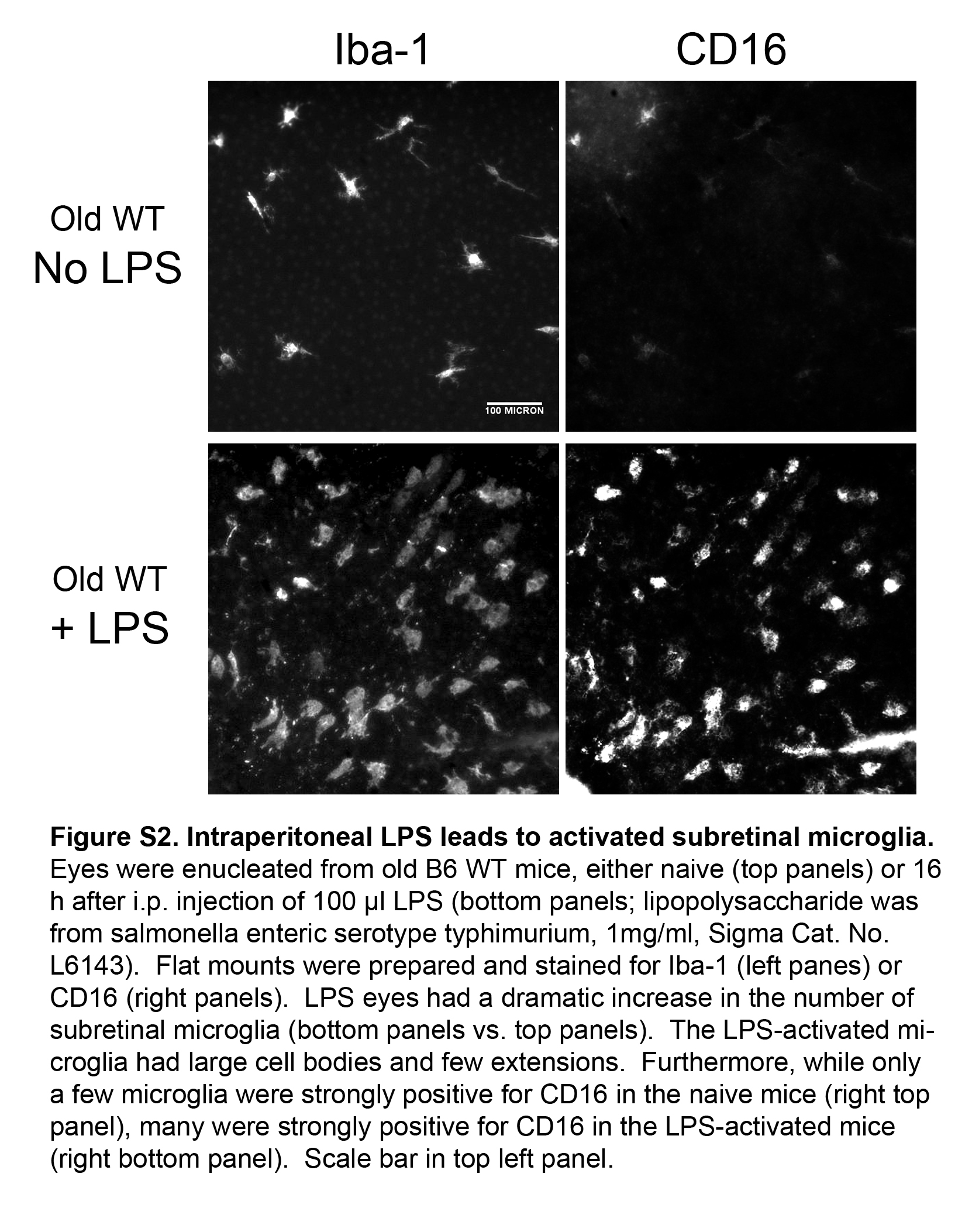

Supplement: Additional file 6: Figure S2. — Intraperitoneal lipopolysaccharides leads to activated subretinal microglia/macrophages. Eyes were enucleated from old B6-wild-type (WT) mice, either naive (top panels) or 16 h after intraperitoneal (i.p.) injection of 100 μl lipopolysaccharides (LPS) (bottom panels; lipopolysaccharide was from salmonella enteric serotype typhimurium, 1mg/ml, Sigma-Aldrich, Inc., St. Louis, MO, USA; Cat. No. L6143). Retinal pigment epithelium (RPE) flat mounts were prepared and stained for ionized calcium binding adaptor (lba)-1 (left panels) or Fcγ III/II receptor (CD16/CD32, abbreviated as CD16, right panels). LPS eyes had a dramatic increase in the number of subretinal microglia/macrophages (MG/MΦ) (bottom panels versus top panels). The LPS-activated MG/MΦ had large cell bodies and few extensions. Furthermore, while only a few MG/MΦ were strongly positive for CD16 in the naive mice (right top panel), many were strongly positive for CD16 in the LPS-activated mice (right bottom panel). Scale bar in top left panel. [file 12974_2014_221_MOESM6_ESM.jpeg]
